# Thrombogenic and Inflammatory Reactions to Biomaterials in Medical Devices

**DOI:** 10.3389/fbioe.2020.00123

**Published:** 2020-03-12

**Authors:** Carlos A. Labarrere, Ali E. Dabiri, Ghassan S. Kassab

**Affiliations:** California Medical Innovations Institute, San Diego, CA, United States

**Keywords:** biomaterials, thrombosis, medical devices, inflammation, tissue factor, C-reactive protein, monomeric C-reactive protein, sulfated glycosaminoglycans

## Abstract

Blood-contacting medical devices of different biomaterials are often used to treat various cardiovascular diseases. Thrombus formation is a common cause of failure of cardiovascular devices. Currently, there are no clinically available biomaterials that can totally inhibit thrombosis under the more challenging environments (e.g., low flow in the venous system). Although some biomaterials reduce protein adsorption or cell adhesion, the issue of biomaterial associated with thrombosis and inflammation still exists. To better understand how to develop more thrombosis-resistant medical devices, it is essential to understand the biology and mechano-transduction of thrombus nucleation and progression. In this review, we will compare the mechanisms of thrombus development and progression in the arterial and venous systems. We will address various aspects of thrombosis, starting with biology of thrombosis, mathematical modeling to integrate the mechanism of thrombosis, and thrombus formation on medical devices. Prevention of these problems requires a multifaceted approach that involves more effective and safer thrombolytic agents but more importantly the development of novel thrombosis-resistant biomaterials mimicking the biological characteristics of the endothelium and extracellular matrix tissues that also ameliorate the development and the progression of chronic inflammation as part of the processes associated with the detrimental generation of late thrombosis and neo-atherosclerosis. Until such developments occur, engineers and clinicians must work together to develop devices that require minimal anticoagulants and thrombolytics to mitigate thrombosis and inflammation without causing serious bleeding side effects.

## Introduction

Venous and arterial thromboses are set in motion by biological processes that activate both *coagulation and inflammation*. In pathological states involving arterial and/or venous thrombosis, the close interrelationship between inflammation and thrombosis becomes apparent. In both arterial and venous thromboses, inflammation can trigger thrombosis and thrombosis can amplify inflammation (Libby and Simon, 2001). Blood-contacting medical devices need to reduce/avoid thrombosis initiated by platelet activation, tissue factor, and contact coagulation factors, thereby limiting generation of proinflammatory components like microparticles released from activated cells (monocytes, endothelial cells), platelets, and/or apoptotic cells. Microparticles harbor proinflammatory molecules like monomeric C-reactive protein (mCRP) and tissue factor that enhance thrombus progression and inflammation. As microparticles play a role in venous and arterial thromboses and are emergent thrombotic disease biomarkers, they may be used to detect biodevices prone to failure. In this review, we will consider: (a) the role of proinflammatory and prothrombotic components like mCRP and tissue factor in atherothrombosis and venous thromboembolism; (b) the participation of proinflammatory and prothrombotic microparticles in atherothrombosis and venous thromboembolism; (c) the role of proinflammatory and prothrombotic factors in blood-contacting device thrombogenesis; and (d) the utilization of contemporary understanding of atherothrombosis and venous thromboembolism to identify future directions in biomaterial research to minimize and prevent thrombosis on blood-contacting devices.

## Mechanism of Thrombus Development and Progression

The normal status of a vessel, being an artery or a vein, is to maintain a well-regulated hemostasis to sustain blood fluidity allowing for rapid thrombus formation to effectively close damaged blood vessels. Hemostatic failure may result in intravascular thrombosis with partial or complete arterial and/or venous occlusion. Arterial thrombosis (atherothrombosis) and venous thromboembolism that involves leg deep vein thrombosis and pulmonary embolism are significant contributors to worldwide disease burden (Raskob et al., 2014), with atherothrombosis being the leading cause of death around the globe (Lozano et al., 2012).

### Biology of Atherothrombosis

**Figure 1:**
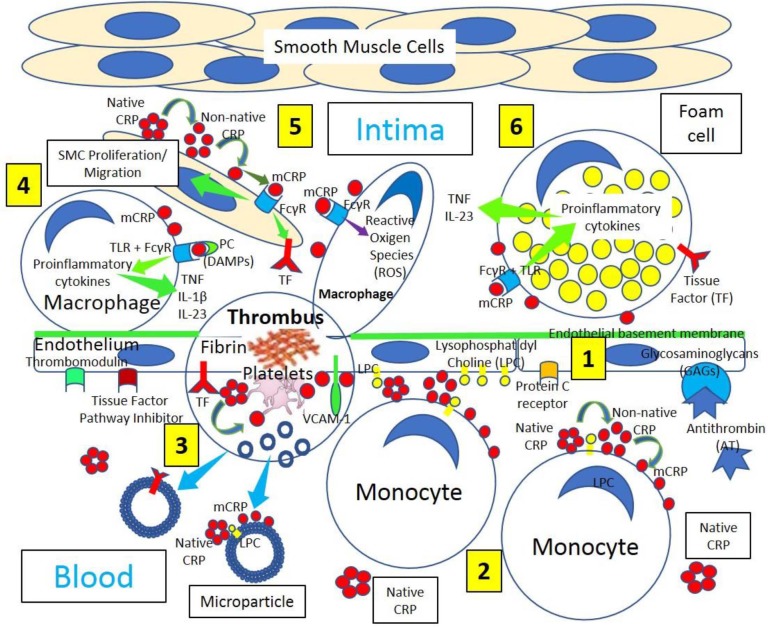
Arterial inflammation and thrombus formation. **[1]** Arterial endothelium normally has antithrombotic properties expressing thrombomodulin, tissue factor pathway inhibitor, protein C receptor, and glycosaminoglycans (GAGs) like heparan sulfate proteoglycans binding antithrombin (AT). **[2]** Blood has circulating native C-reactive protein (CRP) that binds to monocytes through lysophosphatidylcholine (LPC), generating membrane-associated nonnative CRP and monomeric CRP (mCRP). LPC-mediated CRP-binding also occurs on activated endothelium. **[3]** Thrombus formation occurs on activated and damaged arterial endothelium expressing adhesion molecules, like vascular endothelial adhesion molecule-1 (VCAM-1), and tissue factor (TF). Arterial thrombi contain platelets and fibrin, generate mCRP and microparticles containing mCRP and TF. **[4]** Arterial intima in atherosclerotic lesions sustains inflammation, in part, through cytokine (Tumor necrosis factor, TNF; interleukin-1β, IL-1β; and interleukin 23, IL 23) release mediated by Fcγ receptor (FcγR) and toll-like receptor (TLR) activation by CRP complexed to phosphorylcholine (PC) moieties in damage-associated molecular patterns (DAMPs). **[5]** CRP, most probably mCRP, leads to smooth muscle cell (SMC) proliferation/migration upregulating TF and enhanced macrophage reactive oxygen species (ROS) production through FcγR activation. **[6]** Foam cells in atherosclerosis participate in lesion complications and atherothrombosis upregulating TF and generating proinflammatory cytokines following FcγR and TLR activation.

Atherothrombosis is a complex and abnormal chronic inflammatory/immune arterial wall remodeling initiated by the deposition of lipids and oxidative stress followed by recruitment of circulating leukocytes, proliferative responses with atheromatous plaque growth, proteolysis, neo-angiogenesis, apoptosis, calcification, fibrosis, plaque rupture, and thrombosis (Martin-Ventura et al., 2017). While platelets play a central role in arterial thrombogenesis because of fast flow conditions, a thrombus developing on disrupted plaques regularly involves great quantities of fibrin (Figure 1). Platelets, coagulation factors that are part of the extrinsic and intrinsic coagulation pathways, proinflammatory components, hypoxia within the plaque, and abnormalities in blood flow affect formation and propagation of a thrombus. Although a thrombus does not always completely occlude the vessel lumen, its propagation becomes critical in tissues with a terminal vascular bed like the heart and brain triggering cardiac ischemia/infarction and stroke (Asada et al., 2018).

### Pathophysiology of Venous Thrombosis and Thromboembolism

**Figure 2:**
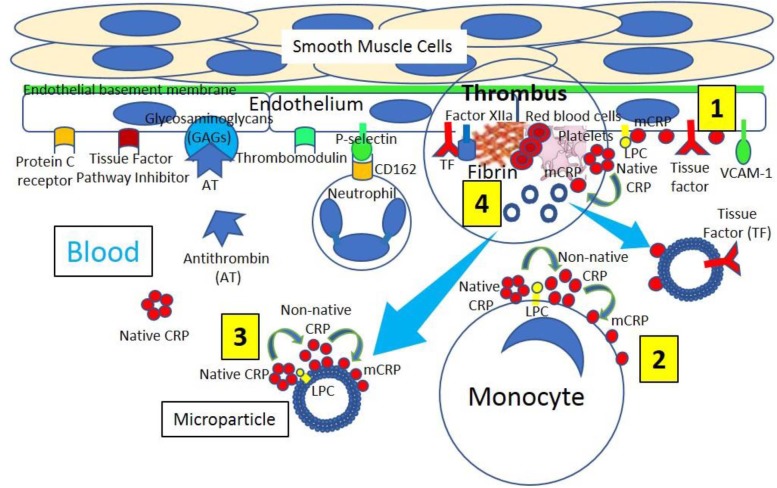
Venous thrombus formation. **[1]** Venous endothelium normally has antithrombotic properties expressing protein C receptor, tissue factor pathway inhibitor, glycosaminoglycans (GAGs) like heparan sulfate proteoglycans binding antithrombin (AT) and thrombomodulin. Activated and damaged endothelium expresses adhesion molecules like P-selectin, that binds P-selectin ligand (CD162) on neutrophils, and vascular endothelial adhesion molecule-1 (VCAM-1); and tissue factor (TF). Activated endothelium generates lysophosphatidylcholine (LPC)-mediated dissociation of C-reactive protein (CRP) into monomeric CRP (mCRP). **[2]** Blood has circulating native CRP that binds to monocytes through LPC, generating membrane-associated non-native CRP and mCRP. **[3]** Venous thrombi generate microparticles from activated cells (monocytes, endothelial cells) or platelets, or from apoptotic cells that bind native CRP through LPC leading to production of non-native CRP and dissociation into mCRP. **[4]** Venous thrombus formation containing platelets and fibrin occurs on activated and damaged venous endothelium expressing TF and participating in Factor XIIa generation. Activated platelets can dissociate native CRP into mCRP and generate microparticles containing mCRP and TF.

**Figure 3:**
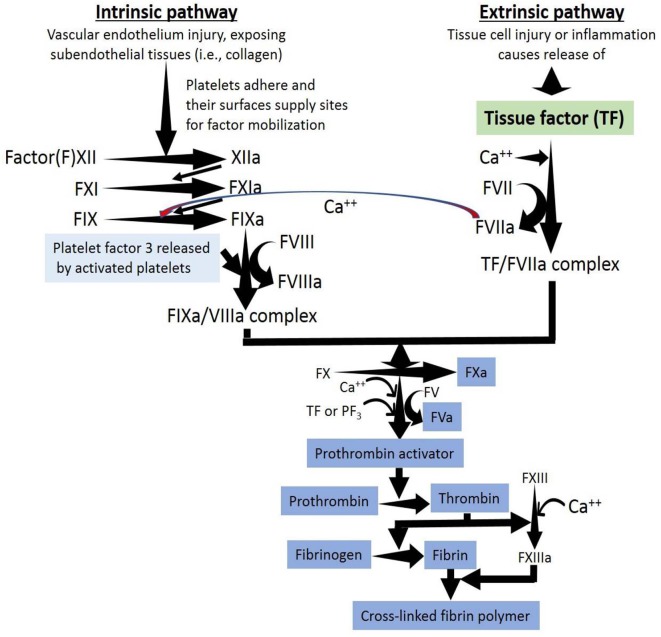
A thrombus is the result of the activation of the extrinsic, intrinsic and common pathways of the coagulation cascade within a vessel. The coagulation cascade is classically divided into three pathways. The tissue factor (extrinsic) and contact activation (intrinsic) pathways both activate the final common pathway of factor X, thrombin and fibrin. Both the TF/factor VIIa and the factor IXa/VIIIa complexes activate FX ending with the generation of fibrin. Factor IX of the intrinsic pathway can be also activated by the TF mechanism.

Venous thromboembolism pathophysiology is traditionally attributed to the Virchow's triad characterized by (a) endothelial injury, (b) venous stasis, and (c) hypercoagulability (Virchow, 1851). An extension of the Virchow's triad describing a mechanistic foundation that facilitates the understanding and grouping of venous thromboembolism causes was recently proposed. Reduced flow and hypoxia, activation of endothelium, components of the innate and acquired immune systems and platelets, as well as type and amount of microparticles and amount of pro- and anticoagulants were described to play a role in venous thrombosis (Figure 2) and venous thromboembolism (Reitsma et al., 2012). Normal endothelium shows the expression of various anticoagulants, among them thrombomodulin, tissue factor pathway inhibitor, endothelial protein C receptor, and heparin-like proteoglycans binding antithrombin (Mackman, 2012), but venous endothelium activated by oxidative stress expresses adhesion molecules recruiting leukocytes and platelets (Figure 2) (Reitsma et al., 2012). Coagulation activation is the principal step in venous thrombosis; tissue factor exposure initiates the extrinsic pathway, while damaged granulocyte released products activate factor XII initiating the intrinsic pathway (Figure 3) (Reitsma et al., 2012). Platelets play a less important role in venous thrombosis than in atherothrombosis, becoming relevant at a later phase, because the initial thrombus is platelet free, while subsequent venous thrombus progression contains platelets (Riva et al., 2015). Thrombin generation may be induced by platelet-mediated activation of both extrinsic and intrinsic coagulation pathways (Reitsma et al., 2012). Inflammation may act upon three key pieces of coagulation:(a) initiation/propagation of the activation of the coagulation cascade, (b) reduction/suppression of physiologic anticoagulant pathways, and (c) impossibility to remove fibrin deposits (Levi et al., 2004).

### Thrombosis and Inflammation and C-Reactive Protein Isoforms

Inflammation-related molecules like native pentameric C-reactive protein (native CRP) contribute to thrombosis since at concentrations commonly found *in vivo* during inflammation (10–100 mg/L), CRP can increase 75 times the tissue factor procoagulant activity in monocytes with concomitant increase in tissue factor antigen levels (Cermak et al., 1993). CRP (24 mg/L) activates inflammation and coagulation by increasing the circulating levels of interleukin-6, interleukin-8, E-selectin, serum amyloid A, type II secretory phospholipase A2, prothrombin F1 + 2, von Willebrand factor, D-dimer, and plasminogen activator inhibitor-1, as previously described (Bisoendial et al., 2005). These effects probably mediated by monocyte/macrophage tissue factor upregulation (Figures 1, 2) may be the result of cell and/or microparticle-related monomeric CRP (mCRP) and non-native pentameric CRP (non-native CRP) generation (McFadyen et al., 2018; Singh and Agrawal, 2019). Native CRP does not participate in thrombogenesis, whereas mCRP induces activation of platelet (de la Torre et al., 2013), platelet adhesion following upregulation of P-selectin (Molins et al., 2008), and thrombus progression under arterial flow conditions (Molins et al., 2008; Badimon et al., 2018); mCRP being identified in platelet aggregates (Figures 1, 2) stimulates additional platelet aggregation (de la Torre et al., 2013). The blockage of glycoprotein IIb–IIIa on activated platelets prevents dissociation of native CRP to mCRP reducing the arterial platelet deposition (de la Torre et al., 2013). The binding of mCRP to phosphorylcholine on activated macrophage and foam cell membranes mediated by concomitant Fcγ receptor and toll-like receptor signaling (Newling et al., 2019) and the presence of mCRP within advanced atherosclerotic plaques seem to play a critical role in the development and progression of a plaque by promoting inflammation, oxidation, smooth muscle cell proliferation, and migration and thrombus formation (Figure 1).

### Pathogenesis of Vascular Thrombosis

Tissue factor is pivotal in initiating inflammation-induced thrombin generation (Figure 3). The complex tissue factor-factor VIIa activates factor X converting it into factor Xa; factor Xa generates thrombin (factor IIa), and thrombin transforms fibrinogen into fibrin (Figure 3). Coagulation activation in deep vein thrombosis is evidenced by higher D-dimer concentrations resulting from massive fibrin generation followed by plasmin-mediated fibrin/fibrinogen degradation (Ghozlan et al., 2015). Following inflammation-induced activation of the coagulation cascade, the three most important anticoagulant pathways (antithrombin, protein C system, and tissue factor pathway inhibitor) become altered (Levi et al., 2004), promoting a prothrombotic state in venous thromboembolism (Riva et al., 2015).

Endothelial cells have antithrombotic properties (e.g., prostacyclin, thrombomodulin, heparan sulfate), participate in the formation of platelet and fibrin thrombi (e.g., von Willebrand factor, tissue factor), synthesize and secrete plasminogen activators and inhibitors, synthesize and secrete angiotensin-converting enzyme, bind hormones, bind lipoproteins, are targets of autoimmune diseases, and directly participate in immune reactions. Endothelial cells are the first barrier between the blood and extravascular space influencing circulation's structural and functional integrity (Jaffe, 1987). The endothelial glycocalyx plays a fundamental vasculo-protective role against thrombosis through the participation of four fundamental anticoagulant pathways: (a) antithrombin, (b) heparin cofactor II, (c) thrombomodulin, and (d) tissue factor pathway inhibitor, with antithrombin being a powerful inhibitor of thrombin and activated factors FXa and FIXa (Reitsma et al., 2007).

The first step in intravascular thrombosis involves tissue factor (Figures 1–3) exposed following vascular injury and/or atherosclerotic plaque rupture. Cell-associated tissue factor is key in atherothrombosis, but discoveries of different circulating pools of tissue factor (microparticle-bound, soluble form) changed the belief that tissue factor activity was only identified in membrane-bound tissue factor (Figures 1, 2). Increased tissue factor activity in circulation enhances the starting thrombogenic stimulus provoked by exposure of vessel wall tissue factor, promoting greater and/or steadier thrombogenesis and more serious clinical complications. Inflammation enhances tissue factor expression and activity facilitating further inflammation by generating proinflammatory fibrin fragments, FVIIa, FXa, and thrombin (Figure 3). Tissue factor biology became more complex since tissue factor also participates in intracellular signaling, proliferation, angiogenesis, and tumor metastasis.

Microparticles, extracellular microvesicles (30–1,000 nm) detached from activated (monocytes, endothelial cells) or apoptotic cells and platelets (Figures 1, 2), harboring numerous cell surface receptors, mRNA, and biological activities, are dramatically increased in thrombosis-associated disorders, express coagulation activators like tissue factor and coagulation inhibitors, bear fibrinolytic properties, play a role in venous and arterial thrombosis and venous thromboembolism, and are emergent biomarkers in thrombotic diseases (Lacroix et al., 2013; Date et al., 2017). Tissue factor-microparticle disruption in inflammation seems to impact monocyte/macrophage-mediated endothelial activation (Date et al., 2017) and circulating tissue factor microparticles (Figures 1, 2); it also participates in venous thromboembolism and disseminated intravascular coagulation (van Es et al., 2015).

### Relationship Between Arterial and Venous Thrombosis

A link between venous and arterial thrombosis is preeminent since (a) both venous and arterial thromboses share risk factors (age, hypertension, obesity, hypertriglyceridemia, diabetes, and metabolic syndrome); (b) several conditions (hyperhomocysteinemia, antiphospholipid antibody syndrome, infections, hormonal treatment, and malignancies) account for both venous and arterial thromboses; (c) patients with venous thromboembolism have greater risk for developing arterial thrombosis; and (d) both venous and arterial thromboses are triggered by biological stimuli concomitantly activating coagulation and inflammation (Prandoni, 2009). Inflammation, with its associated prothrombotic environment, could explain the reported connection between arterial and venous thromboembolism events (Riva et al., 2015). Traditionally, arterial and venous thromboses were considered as two dissimilar diseases: (a) arterial thrombi, mainly composed of platelets and fibrin (white thrombi), mostly occur in areas of atherosclerotic plaque rupture with high shear stress (Figure 1), while venous thrombi, mainly comprised of red blood cells and fibrin in addition to activated platelets (red thrombi), mostly occur at sites of low blood flow and shear rates and around intact endothelial walls (Figure 2) (Koupenova et al., 2017). However, recently it was suggested that atherothrombosis and venous thromboembolism have similar causes including inflammation (Riva et al., 2015).

## Thrombosis and Inflammation Induced by Biomaterials

### Pathogenesis of Thrombosis and Inflammation in Biomaterials

**Figure 4:**
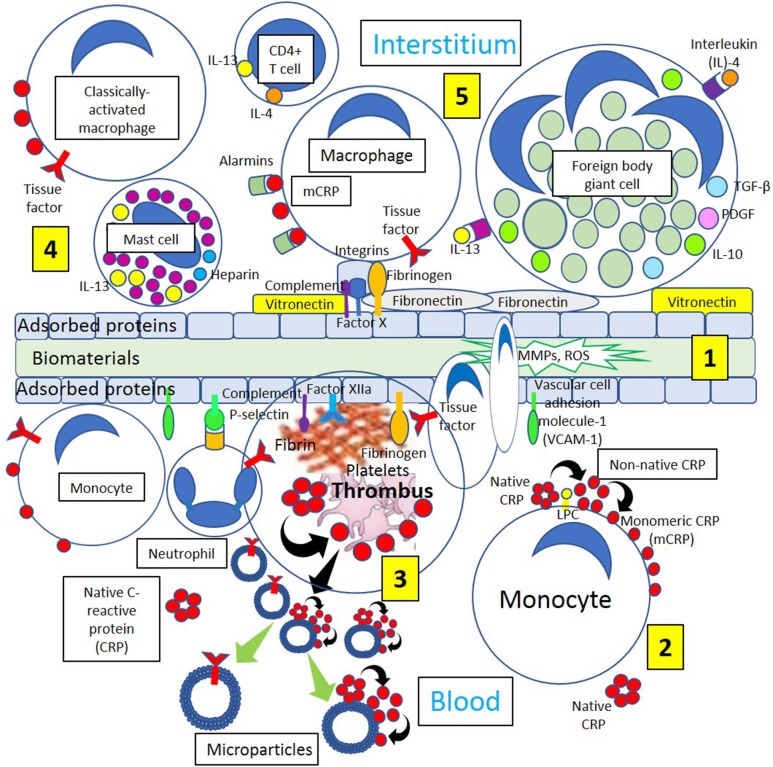
Different biological processes occurring on the surface and around biomaterials. **[1]** Immediately after the first contact with the recipient's tissues, blood proteins, and interstitial fluids become adsorbed to the surface of the biomaterials. Adsorption of circulating proteins (adhesion molecules vascular cell adhesion molecule-1 [VCAM-1] and P-selectin, complement components, and fibrinogen, among other proteins) to biomaterials facilitates neutrophil and macrophage biomaterial cell adhesion. During interstitial generation of foreign body giant cells, macrophages bind to different biomaterial adsorbed proteins including vitronectin, complement components, Factor Xa, fibrinogen, and fibronectin through different integrins present on the surface of macrophages and other inflammatory cells. **[2]** Blood has circulating native C-reactive protein (CRP) that binds to monocytes through lysophosphatidylcholine (LPC), generating membrane-associated nonnative CRP and monomeric CRP (mCRP). **[3]** Thrombi are formed through activation of coagulation factors (Fibrinogen, Factor XIIa) adsorbed to the biomaterials and tissue factor (TF) from inflammatory cells (neutrophils, monocytes). Platelet activation dissociates native CRP into mCRP. Activated and apoptotic cells, as well as platelets, generate microparticles that can dissociate native CRP into mCRP and contain TF. Cells from circulation attach to the protein surface and penetrate the biomaterial producing matrix metalloproteinases (MMPs) and reactive oxygen species (ROS) initiating inflammation. **[4]** Classically activated macrophages, CD4+ T-cells and mast cells participate in inflammatory processes releasing inflammatory cytokines interleukin (IL)-4 and IL-13, and macrophages generate mCRP promoting inflammation. **[5]** Endogenous pattern-associated molecular pattern equivalents **(**alarmins) through pattern recognition receptors like mCRP, contribute to inflammation, and integrin-mediated macrophage-binding to vitronectin and fibronectin and other adsorbed proteins lead to generation of foreign body giant cells participating in inflammation.

Based on the antithrombotic properties of the normal vessels, biomaterials need to be intensely bioengineered in order to provide surfaces that minimize coagulation activation and thrombus formation in any vessel, either an artery or a vein (Franz et al., 2011). Biomaterials when implanted in living tissues always initiate a healing response to the foreign body. The purpose is to integrate the biomaterial to the body avoiding development of chronic inflammation that eventually will end with implant failure. Immediately after the first contact with the recipient's tissues, proteins in the blood and interstitial fluids attach to the surface of the biomaterial (Figure 4). This layer of adsorbed proteins regulates the activation of coagulation, complement, platelets, and immune cells and conducts their interaction leading to matrix formation and an inflammatory response (Gorbet and Sefton, 2004; Wilson et al., 2005; Franz et al., 2011). Surface activation and auto-activation of Factor XII bound to adsorbed proteins and the presence of Factor VII on the cell membranes of monocytes, macrophages, and other injured cells added to platelet adhesion and activation initiate coagulation activation ending with the generation of thrombin and fibrin and thrombus formation. Fibrinogen also spontaneously adsorbs to biomaterial surfaces and is capable of activating monocyte/macrophages through integrins binding initiating the inflammatory response (Figure 4). The activation of complement is always associated with complement binding to the adsorbed protein layer on the biomaterial (Nilsson et al., 2007; Franz et al., 2011). Activation of the complement cascade generates large amounts of C3a and C5a at the implantation site (Andersson et al., 2005). Both C3a and C5a participate in setting in motion inflammatory responses at implantation sites mediated by numerous effector functions that include triggering degranulation of mast cell, intensification of vascular permeability, attraction and activation of leukocytes and monocytes, and induction of leukocyte reactive oxygen species release (Sarma and Ward, 2011). Complement activation supports platelet adhesion and activation on biomaterial surfaces and promotes tissue factor expression on leukocytes and monocytes further stimulating the coagulation cascade (Franz et al., 2011) (Figure 4). Extracellular matrix proteins like vitronectin and fibronectin also attach to biomaterial surfaces (McFarland et al., 2000; Keselowsky et al., 2007) and are paramount in managing the inflammatory response to biomaterials. Vitronectin and fibronectin can stimulate the fusion of macrophages generating foreign body giant cells on the surface of the biomaterial, although only vitronectin supports macrophage adhesion and fusion (Keselowsky et al., 2007; McNally et al., 2008). Neutrophilic polymorphonuclear leukocyte adhesion to biomaterial surfaces can be mediated by P-selectin participating in the pathogenesis of inflammation and fibrosis induced by biomaterials (Tang et al., 2002). Biomaterials are associated with receptor-ligand interactions that activate immunocompetent/inflammation-related cells. Endogenous equivalents of pathogen-associated molecular patterns known as alarmins (heat shock proteins, high mobility group box 1, adenosine triphosphate, uric acid) are recognized by innate immune cells (macrophages, dendritic cells) through pattern recognition receptors like CRP (Figure 4), scavenger receptors, toll-like receptors, and C-type lectins contributing to inflammation and immunity (Franz et al., 2011).

**Figure 5:**
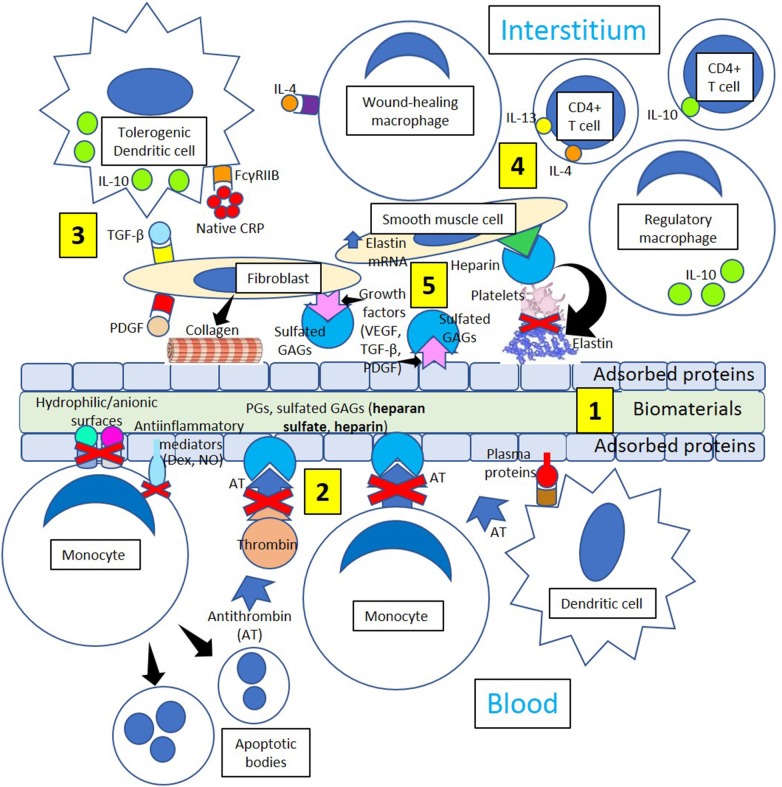
Minimizing thrombosis and inflammation in biomaterials. **[1]** Biomaterial adsorbed plasma proteins facilitate adhesion of dendritic cells that become interstitial tolerogenic dendritic cells. **[2]** Proteoglycans (PGs) and sulfated glycosaminoglycans (GAGs, heparan sulfate, heparin) bind antithrombin and inhibit thrombin generation and monocyte migration. Hydrophilic/anionic surfaces promote decreased adhesion, an anti-inflammatory response by adherent monocytes and increased apoptosis, reducing inflammation. Anti-inflammatory mediators like dexamethasone (Dex) and nitric oxide (NO) further promote resolution of inflammation. **[3]** Tolerogenic dendritic cells could induce a tolerogenic effect on biomaterials by releasing interleukin-10 (IL-10) following native CRP stimulation through Fc gamma receptor IIB (FcγRIIB), so acting as a stopper on T cell mediated immunity shifting the balance toward tolerance. Profibrotic molecules like transforming growth factor-beta (TGF-β) and platelet-derived growth factor (PDGF) activate fibroblasts, and activated fibroblasts synthesize and deposit collagen leading to biomaterial encapsulation. **[4]** Tolerogenic dendritic cells, wound-healing macrophages, regulatory macrophages, CD4+ T-cells, smooth muscle cells, and fibroblasts participate in the healing process through the mediation of anti-inflammatory cytokines like interleukin (IL)-10 and collagen and elastin formation. **[5]** Interaction between growth factors VEGF, TGF-β and PDGF and sulfated GAGs in the interstitium and on the surface of adsorbed proteins inhibit platelet activation and thrombus formation. Heparin improves cell activity around implants influencing biomaterial integration and enhancing the inhibition of platelet activation mediated by elastin derivatives.

The persistency of inflammatory stimuli at the biomaterial implantation site perpetuates the immune response in which monocytes/macrophages are fundamental participants (Figure 4). Monocytes reaching the implantation site differentiate into different subsets of macrophages that participate in inflammation, wound healing, and tissue regeneration. These subsets are at present classified into classically activated, regulatory, and wound healing macrophages (Mosser and Edwards, 2008). Classically activated macrophages are mostly induced by interferon-γ released by T helper 1 or natural killer cells and by tumor necrosis factor-α released by antigen presenting cells. Wound-healing macrophages are produced following the release of interleukin-4 by T helper 2 cells, basophils, mast cells, and granulocytes (Figure 5). Regulatory macrophages place a limit on inflammation and reduce immune responses through the release of high levels of interleukin-10, a very potent immunosuppressive cytokine (Franz et al., 2011) (Figure 5). Macrophages attached to a foreign biomaterial typically have a classically activated phenotype secreting inflammatory cytokines, reactive oxygen species, and proteolytic enzymes and showing high phagocytic capabilities.

Production of interleukin-4 and interleukin-13 by CD4+ T cells and most probably, sustained generation of interleukin-13 by mast cells as a chronic inflammatory response to biomaterials, stimulate macrophage fusion and development of foreign body giant cells (Figure 4). Furthermore, the binding of vitronectin to adsorbed proteins onto biomaterials supports macrophage adhesion and fusion (Franz et al., 2011) (Figure 4). Macrophage-derived proteolytic enzymes participating in foreign body reaction, such as matrix metalloproteinases and reactive oxygen species released by foreign body giant cells, play a part in the degradation and resorption of biomaterials (MacLauchlan et al., 2009; Franz et al., 2011) (Figure 4). Although foreign body giant cells switch to a more alternative anti-inflammatory macrophage phenotype over time mainly producing interleukin-10 and monocyte chemoattractant protein-1, among others, to promote wound healing, they still produce proinflammatory molecules, reactive oxygen species, and degradative enzymes (Franz et al., 2011).

### Minimizing Thrombosis and Inflammation in Biomaterials

Foreign body giant cells formed following interleukin-4 stimuli (Figure 4) may release profibrotic molecules like transforming growth factor-beta and platelet-derived growth factor activating fibroblasts, and activated fibroblasts synthesize and deposit collagen leading to biomaterial encapsulation (Figure 5). Dendritic cells are fundamental players of both innate and adaptive immunity and could induce a tolerogenic effect on biomaterials by releasing interleukin-10 (Franz et al., 2011) following native CRP stimulation (Figure 5). It has been recently proposed that native CRP down-regulates dendritic cell development and activation/maturation through Fc gamma receptor IIB (FcγRIIB), so it acts as a stopper on T cell mediated immunity, shifting the balance toward tolerance (Jimenez et al., 2018) (Figure 5). Inducing tolerogenic capabilities of biomaterials appears a promising strategy to down-regulate the host immune responses and resolve inflammation.

T-cell-macrophage interactions promote macrophage adhesion and fusion (Brodbeck et al., 2005; Franz et al., 2011), and the initial proinflammatory response to biomaterials releasing interleukin-1β and tumor necrosis factor-α switches over time toward the release of interleukin-10, matrix metalloproteinase-9, and tissue inhibitors of matrix metalloproteinases 1 and 2 facilitating extracellular matrix remodeling in wound healing (Chang et al., 2008). The extracellular matrix structure provides a frame for cellular structure, and the presence of fibrin, collagen, and elastin provides strength and elasticity (Figure 5). The protein frame is embedded in a negatively charged matrix of sulfated glycosaminoglycans (GAGs) like heparan sulfate and others that interact with growth factors [platelet derived growth factor (PDGF), vascular endothelial growth factor (VEGF), and transforming growth factor-β (TGF-β)], reducing growth factor sensitivity to enzymatic cleavage and developing a distinct cellular environment that regulates the regeneration of tissues (Chen et al., 2007). The extracellular matrix also modifies cell behavior by allowing interactions with other cells and extracellular components, proteolytic enzymes, and signaling molecules associated with tissue repair, host responses to biomaterials, and biomaterial consolidation (Franz et al., 2011).

Although inert biomaterials remain practically unchanged and tolerated by the host due to fibrous tissue encapsulation, regulation of cell adhesion to the protein layer adsorbed to biomaterials (Dinnes et al., 2007) may change cell responses leading to improved wound healing. Biomaterial surfaces modified with polyethylene glycol could prevent cell attachment and activation (VandeVondele et al., 2003) facilitating proper wound healing. Facilitating the binding of biomaterials to growth factors, such as VEGF, TGF-β, and PDGF, can control adhesion, migration, proliferation, and differentiation of fibroblasts, smooth muscle cells, and other cells improving wound healing (Barrientos et al., 2008). Furthermore, growth factors could be involved in the maintenance of an antithrombotic environment through the mediation of sulfated glycosaminoglycans (Figure 5), promoting linkage to interleukin-10 (Salek-Ardakani et al., 2000) and enhancing cell activity around biomaterials influencing integration (Franz et al., 2011). Then, modifying biomaterials in such a way to allow coagulation and specific cell responses to be beneficial for implant integration and performance is a novel approach in biomaterial research.

## Mathematical Models of Thrombosis

Arterial and venous thromboses are complex processes involving multiple variables. Biophysical mathematical models are useful to capture the complexity of the process allowing predictions *in vivo* where the boundary conditions at the blood-device interface are not easily measurable. The last two decades showed an exponential growth in thrombogenesis studies using computational tools and *in vitro* experiments. However, the complete mechanism underlying thrombus formation and hemostasis is not known. There is no single model predicting thrombus physiological and mechanical properties, although models and methods used are diversified into different spatiotemporal scales (Yesudasan and Averett, 2018).

A mathematical model to initiate venous thrombosis caused by slow flow and venous endothelial cell activation in the absence of noticeable mechanical endothelial cell layer disruption revealed that a strong thrombin generation depends on the tissue factor density on activated endothelial cells and the thrombomodulin concentration and degree of heparan-sulfate accelerated antithrombin cell activity (Elizondo and Fogelson, 2016). The model considered all tissue factor coagulation pathway reactions through the formation of fibrin, incorporated the accumulation of blood cells on activated endothelial cells, and accounted for flow-mediated delivery and removal of coagulation proteins and blood cells from the reaction site and the activity of major inhibitors like heparan-sulfate-mediated accelerated antithrombin activity and activated protein C. The model predicted collaboration among the inhibitors and demonstrated that the rate and extent of activated monocytes, platelets, and microparticle accumulation contributing to coagulation heavily influence whether strong thrombin generation occurs. The slow rate of cell accumulation in venous thrombosis is one reason its progress is so much slower than that of atherothrombosis initiated by plaque rupture.

This is the first mathematical model of slow initiation of venous thrombosis as occurring in the pockets behind dysfunctional venous valves (Elizondo and Fogelson, 2016), incorporating many known biological features and allowing a quantitative examination of their roles in venous thrombosis. The model, however, has many limitations. First, it is based on a simple treatment of flow that may not adequately represent the transport rates of cells and proteins to and from the valve pocket depths. Second, it assumes that all circulating activated blood cells and microparticles contribute to venous thrombosis as a composite homogeneous population. Third, it has marked unpredictability regarding thrombomodulin and heparan sulfate expression levels and the degree to which heparan sulfate can accelerate antithrombin-mediated inhibition of coagulation proteases. Fourth, it includes fibrin dynamics only to the extent of fibrin monomer formation and the binding/unbinding of thrombin to fibrin. Fifth, computational results for most simulations extend only to 24 h, albeit the dynamics become steadily long before this time point. Finally, the model does not consider many interactions (formation of extensive and dense fibrin gel, red blood cell entrapment, capture of neutrophils, neutrophil release from extracellular nets) involved in venous thrombus propagation following its initial stage that would likely change subsequent dynamics. Despite the limitations, the mathematical model is hypothesis-generating. Models naturally evolve with increasing complexity as (a) more information regarding the types and characteristics of the cells and microparticles contributing to venous thrombosis becomes available, and (b) more knowledge about flow and the inhibitory environment in venous valve pockets becomes known.

An Eulerian-Lagrangian model was recently developed (Yazdani et al., 2017) in order to predict the shape and growth of a thrombus, where the motions of Lagrangian platelets were coupled with background blood flow utilizing a force coupling method taking account of platelet adhesion to the site of injury and to each other by a shear-dependent Morse potential that was calibrated with experimental data for different shear conditions. Simulation results showed a good agreement with the experiments performed for a wide range of shear rates, which suggested that the model is suitable for venous thrombosis and embolization as well as arterial thrombosis.

Recently, it was suggested (Yesudasan and Averett, 2018) that trends in computational modeling over the years show that simpler models like single component continuum models are becoming redundant and are increasingly replaced with discrete particle methods like dissipative particle dynamics and multiscale modeling (Zhang et al., 2016). The continuum level methods are still suitable for predicting top level behavior based on empirical data, i.e., the use of Navier-Stokes equations coupled with convection diffusion reaction equations to investigate blood flow dynamics. Two-way coupling of the Navier-Stokes equations with convection diffusion reaction or other methods is superior to one-way coupling in many aspects like accurate flow distribution of blood and thrombus deformations due to high shear flow. A class of multiscale methods at different length and time scales is a promising new approach. Multiscale blood thrombus models can simulate thrombus dynamics, biochemical reactions, factor concentrations, and other parameters. Multiscale models have cascading submodels that can serve as independent models to predict specific characteristics such as: (1) thrombin cleaving fibrinogen, (2) attachment of fibrin to von Willebrand factor, and (3) thrombus solid-liquid interactions. A good multiscale model can simulate the bulk behavior of blood-like diffusivity, viscosity, thrombus mechanical properties under shear flow, and dynamic properties to simulate lysis and thrombus rupture based on molecular level mechanics. Table 1 shows a summary of strengths and limitations of different scales of modeling (Yesudasan and Averett, 2018).

**Table 1:** Strengths and weaknesses of various computational methods.

**Method**	**Advantage**	**Weakness**
System level	Good agreement with experiments	Missing spatiotemporal information
Continuum	Easy to setup Less computational power	Molecular and microscopic details are missing; Need constant default rate equations to account for blood factors
Dissipative particle dynamics	Can work with complex geometries	Computationally expensive for big systems
Multiscale	Detailed information of system included; fine-tuned information at any scale can be included to improve the modeling; reliable simulation results of system under consideration	Complex to model. Needs careful designing of algorithms to solve at different scales. Computationally expensive

In order to predict thrombus formation over time, a hemodynamics-based model to study thrombosis in Type B aortic dissection under realistic flow conditions and different idealized aortic dissections that uses two variables like local shear rates and residence time was recently described (Menichini and Xu, 2016). The data showed that smaller tears could increase thrombosis likelihood due to increased shear stress on the tears, promoting the activation of platelets, and could support the formation of extended recirculation zones. The modeling outcome resulted in good qualitative agreement with *in vivo* observations, where it might be feasible to predict thrombosis in realistic geometries.

Mathematical modeling of blood coagulation and clot formation under flow in normal and pathological conditions was recently described (Bouchnita, 2018). The development of thrombosis in patients with antithrombin deficiency and inflammatory diseases was investigated in order to determine the antithrombin-related thrombosis threshold and quantitatively evaluate the effect of inflammatory cytokines on the coagulation process. A multiscale modeling was applied to blood loss recovery after bleeding focusing on erythropoiesis and the production of hemoglobin. The thrombosis risk in cancer (multiple myeloma in particular) and HIV patients was also evaluated. The model describes the progression of multiple myeloma and its intraclonal heterogeneity. Tumor growth dynamics were simulated during chemotherapy in order to quantify the prothrombotic effects following treatment of multiple myeloma. To predict the risk of thrombosis in HIV-infected patients, a different model focused on the dynamics of HIV infection in lymph nodes. The effects of anticoagulant drugs were evaluated quantitatively by incorporating pharmacokinetics-pharmacodynamics models of those treatments into the blood coagulation model to predict the therapeutic window that should be pursued for individual patients.

In summary, mathematical models of thrombosis can integrate numerous associated biological processes and are hypothesis-generating. The current trend of thrombosis modeling efforts is focused on multiscale methods to understand the underlying mechanisms of thrombosis and to highlight new basic experiments and clinical therapies.

## Blood Compatibility Challenge With Medical Devices

There are several commercially available medical devices implanted in the heart and arteries (e.g., left ventricular assist device, intra-aortic balloon pump, stents, grafts) and veins (e.g., inferior vena cava filter, vein patch or graft) to restore and maintain homeostasis.

These devices must meet various requirements for a patient's long-term use. We will focus on blood compatibility with device “foreign” surfaces. Although normal endothelium is thromboresistant, foreign surfaces have no such safeguards. Thrombosis is the result of flow disturbance and surface biocompatibility (Jaffer et al., 2015).

Medical devices stimulate coagulation through the activation of a series of interconnected processes including adsorption of proteins and adhesion of platelets and leukocytes, among others. The growth rate of thrombosis on implanted surfaces in the veins may be greater than in the arteries due to lower blood velocity and higher residence times in the veins. Advancements in the hemodynamics of medical devices as well as advances in biomaterial developments have the potential to decrease platelet activation and activation of different factors of the coagulation system (i.e., factor XII) (Jaffer et al., 2015).

Protein adsorption upon blood contact with a device's foreign surface is the starting event that leads to thrombus formation. Adsorbed proteins on the biomaterials can form a 2- to 10-nm-thick surface monolayer, and surface protein concentrations can become 1,000-fold higher than in the plasma (Wilson et al., 2005). Surface adsorption is often reversible, and the composition of absorbed proteins varies over time, a phenomenon called “the Vroman effect” (Hirsh et al., 2013). This dynamic change in the composition of the protein monolayer is predominantly evident on negatively charged hydrophilic surfaces (Turbill et al., 1996) and appears to be flow independent (Ortega-Vinuesa et al., 1998). The activation of platelets on biomaterials occurs through platelet adhesion to biomaterials-adsorbed proteins (mainly fibrinogen) and indirectly via biomaterials-induced activation of the coagulation cascade and other systems. The activation of platelets takes place with or without adhesion to a biomaterial surface (Hanson et al., 1980; Cholakis et al., 1989). Cells, like neutrophils and monocytes, may adhere or become activated on the surface of biomaterials, and adhesion to the biomaterials may be mediated via adsorbed proteins (e.g., fibrinogen, fibronectin). Cell-associated released products cause local tissue damage, recruitment, and further inflammatory cell activation (Jaffer et al., 2015).

Several strategies were employed to mitigate thrombosis including the use of antiplatelet drugs (e.g., aspirin, clopidogrel) or anticoagulants (e.g., warfarin). Modification of material surfaces to minimize defense reaction or localize specific anticoagulant release can have clinical benefits, so medical device manufacturers have concentrated on the prevention of protein and cell adsorption, avoidance of thrombin generation and fibrin formation, as well as avoidance of platelet activation.

### Inhibition of Protein and Cell Adsorption

This phenomenon is based on a physical principle where protein adsorption is facilitated by electrostatic and hydrophobic interactions between the adsorbed protein and an external surface. Different materials have been developed to coat surfaces minimizing these interactions. Some of these include albumin, poly(ethylene oxide), and pyrolytic carbon.

#### Albumin

Albumin induces less platelet adhesion (Kim et al., 1974; Park et al., 1986) compared with fibrinogen and globulins and makes it suitable as a thromboresistant coating. Surfaces modified with long aliphatic chains increase the binding of endogenous albumin or warfarin, which strongly binds to albumin (Eberhart et al., 1987; Nelson et al., 1996) and show reduced adhesion of platelets and leukocytes *in vitro* (Kottke-Marchant et al., 1989). However, when a glutaraldehyde cross-linked albumin coating was applied to Dacron grafts, it failed to enhance performance in animals and humans compared with the uncoated grafts (Al-Khaffaf and Charlesworth, 1996; Marois et al., 1996).

#### Poly(ethylene oxide) (PEO)

Hydrophilic ether oxygen in the structural repeat unit of PEO leads to a liquid-like surface with mobile molecular chains. This property of PEO seems to be responsible for lower protein and cell adsorption compared with other polymers (Lee et al., 1995). Several methods have been used to coat biomaterial surfaces with PEO, including bulk modification, covalent grafting, and physical adsorption, but animal studies are inconsistent, and there are no human studies although it has been demonstrated that most PEO-modified surfaces are resistant to protein and cell binding *in vitro*.

#### Pyrolytic Carbon

Medical devices, such as stents, mechanical heart valves, and vascular grafts, have been coated with pyrolytic carbon films manufactured by chemical vapor deposition and used in human studies. Human clinical trials have shown that long-term performance of carbon-coated vascular grafts and coronary stents is similar to uncoated grafts (Kim et al., 2005; Sick et al., 2005) despite the fact that there is less platelet adhesion and spreading on carbon-coated than on uncoated surfaces *in vitro* and in animal studies. Although coating is used for contemporary bileaflet mechanical heart valve components, patients with mechanical valves continue to necessitate lifelong anticoagulation to eliminate or avoid thromboembolic complications.

### Inhibition of Thrombin Generation and Fibrin Formation

Since endothelial cells possess anticoagulant properties that make them non-thrombogenic, seeding biomaterial prosthetic surfaces with endothelial cells and grafting antithrombotic substances onto the biomaterial surface were considered. This process is challenging because of issues associated with the sourcing of the cells, cell stability, cell viability, and cell functionality. Biomaterials introducing bioactive molecules like corn trypsin inhibitor, heparin, thrombin inhibitors like hirudin, bivalirudin, argatroban, or thrombomodulin onto the surfaces were generated (Jaffer et al., 2015). Only corn trypsin inhibitor and heparin will be briefly described since they seem to be more promising in avoiding thrombosis.

#### Corn Trypsin Inhibitor

Since the adsorption of proteins and activation of FXII on the surface of biomaterials are the root cause of thrombin generation and fibrin formation (Jaffer et al., 2015), developing surfaces that resist protein deposition and also inhibit FXIIa through polyethylene glycol-corn trypsin inhibitor conjugate coating (attaching a preformed conjugate; or polyethylene glycol and corn trypsin inhibitor sequentially) is an attractive approach (Alibeik et al., 2012). Both surface attached polyethylene glycol and polyethylene glycol-corn trypsin inhibitor conjugate reduced fibrinogen adsorption. Polyethylene glycol-corn trypsin inhibitor conjugate but not polyethylene glycol coating also inhibited FXII autoactivation, attenuated the activation of FXI mediated by FXIIa, reduced the generation of thrombin, and prolonged the clotting time of recalcified plasma (Alibeik et al., 2012). It has been demonstrated that polyethylene glycol-corn trypsin inhibitor conjugate-coated catheters remained patent 2.5-fold longer than uncoated catheters, catheters coated only with polyethylene glycol, and catheters coated with a polyethylene glycol-albumin conjugate (Yau et al., 2012). Therefore, polyethylene glycol-corn trypsin inhibitor conjugate coating has the potential to attenuate deposition of proteins and inhibit activation of FXII on blood-contacting devices.

#### Heparin

Heparin or covalent heparin-antithrombin complexes have been immobilized on the biomaterial surface (Klement et al., 2006). Randomized clinical trials have demonstrated that the angiographic and clinical endpoints were similar with heparin-coated and bare metal coronary stents (Vrolix et al., 2000; Wöhrle et al., 2001), and neither covalent nor ionic-bonded heparin-coated cardiopulmonary bypass circuits reduced the generation of thrombin, altered the consumption of platelets, or decreased the requirements for postoperative transfusion when compared with uncoated circuits, questioning the use of heparin-coated biomaterials (Edmunds and Colman, 2006). However, numerous studies have shown that keeping the antithrombin-mediated anticoagulant activity of heparin, catalyzing clotting factor inhibition as a covalently immobilized surface coating on a variety of medical devices, is paramount to the design of a functional heparin surface, and functionally active non-eluting heparin bonding remains a viable and effective approach for enhancing the hemocompatibility of medical devices (Biran and Pond, 2017).

Despite decades of effort, small artery grafts (diameter <4 mm) in peripheral circulation that functionally arterialize have not been possible primarily due to thrombosis (Lu et al., 2019). Recently, Lu et al. (2019) constructed a 0.7-mm-diameter graft from swine pulmonary visceral pleura (PVP) and implanted it into rat femoral arteries up to 24 weeks. The total graft patency was 86% in the 24-week period. The neo-endothelial and -medial layers were assembled in the grafts as evidenced by biomarkers for endothelial cells, smooth muscle cells, and extracellular matrix. Agonists-induced vasoconstriction and endothelium-dependent vasodilation were apparent at 12 weeks and amplified at 24 weeks. The high small graft patency suggests that PVP is a promising prosthetic biomaterial for vessel reconstructions, but translational large animal studies are still needed.

## Thrombosis in Medical Devices

We will break down the discussion based on where the device is implemented. It will be arterial system (heart included) and the venous system.

### Devices in the Arterial System

#### Coronary Artery Bypass Grafting

Coronary artery bypass grafting remains an essential procedure to treat patients with multivessel coronary artery disease, especially in diabetic patients. Coronary artery bypass grafting remains key to treat multivessel coronary artery disease. Over 400,000 US patients/year undergo coronary artery bypass grafting (McKavanagh et al., 2017). Although using internal thoracic artery grafts is ideal, often saphenous vein grafts are utilized but have considerable adverse outcomes and mortality (McKavanagh et al., 2017). Saphenous vein graft failure occurs early, intermediate, and late post-surgery. Early failure (<1 month) relates to surgery, thrombosis, hypoxia, proinflammatory, and procoagulant activation, pressure-related increased venous diameter, turbulent blood flow, and shear stress promoting vessel patency reduction. Intermediate (1 month to 1 year) failure is due to progressive neo-intimal hyperplasia and graft atherosclerosis. Late (>1 year) graft failure is due to progressively diffuse and concentric atherosclerosis with thrombosis, myocardial infarction, and death (McKavanagh et al., 2017). Patients without viable veins for autologous grafting need alternative options, like tissue engineering, especially for (a) more than 30% of patients with multiple plaques and (b) pediatric patients needing grafts growing with them. Although tissue-engineered grafts convert into mature vessels through inflammation-mediated remodeling (Roh et al., 2010), they worsen due to thrombosis, intimal hyperplasia, and restenosis caused by uncontrolled chronic inflammation. Optimizing scaffolds to improve the prospects of current tissue engineered vascular graft technologies will advance the field of cardiovascular surgery (Matsuzaki et al., 2019).

#### Stents

Although percutaneous coronary intervention and stent placement revolutionized medicine, arterial injury, neointimal hyperplasia, restenosis, and repeated revascularizations develop in up to one third of patients (Serruys et al., 2001). Stent thrombosis causes myocardial infarction and death in up to 80% (Taniwaki et al., 2016). Thrombosis in bare-metal and drug-eluting stents is associated with early major edge dissections, stent under expansion and insufficient platelet inhibition, plus chronic inflammation and slow arterial healing during late follow-up (Stefanini and Holmes, 2013).

The plenitude of different drug-eluting stents requires knowledge about their characteristics to align the advantages and disadvantages of the stent with the requirement of each unique lesion. Stent performance depends strongly on lesion characteristics. Arterial response to stent implantation is complex, and thus preclinical (i.e., animal) studies are key to predict performance in humans. There is not one ideal stent, but different stents are better suited for different situations. For example, small diameter stents have been designed for the treatment of narrow vessels (<2.5 to 3.0 mm). Since real-world experience showed frequent off-label usage, there has been a broadening of the indications for drug-eluting stent placement. Bare-metal stents are not outdated, but drug-eluting stents have largely replaced them. It has been argued that practice adaption and application of drug-eluting stents have been too fast. Bare-metal stents are still used in about 20% of percutaneous coronary interventions. Bare-metal stents may be preferable in large vessel lesions with acute myocardial infarction. Bare-metal stent device costs are lower than drug-eluting stent device costs. Comparing costs requires considering long-term outcomes that favor drug-eluting stents because of less reinterventions and thus improved cost-benefit analyses (Senst et al., 2019). Mechanisms in very late stent thrombosis occurring beyond 1-year post-drug-eluting stents are not well-defined, and therapies are unsatisfactory. Although newer-generation drug-eluting stents reduced very late stent thrombosis risk to bare-metal stent levels, the accumulated long-term risk is still notable (Taniwaki et al., 2016). Stent thrombosis criteria are based on the time of occurrence (i.e., early, ≤ 1 month; late, >1 month to ≤ 1 year; or very late, >1 year) and the degree of diagnostic certainty (i.e., definite, probable, or possible) (Stefanini and Holmes, 2013). Non-degradable synthetic vascular prostheses promote inflammation and thrombosis, intimal hyperplasia, and high failure rate (Chlupác et al., 2009). Bare-metal stents provoke intense intimal hyperplasia that leads the way to in-stent restenosis in 15–60% of patients within 1–2 years (Chen et al., 2006). Although drug-eluting stents limit early intimal hyperplasia, late-stent thrombosis, late catch-up phenomenon, and neo-atherosclerosis contribute to late failure (Park et al., 2012). Elevated hsCRP predicted increased risks of drug-eluting stent-related thrombosis, death, and myocardial infarction (Park et al., 2009); CRP had a high predictive value in atherosclerotic thrombotic events after drug-eluting stent implantation (Park et al., 2009; Luan and Yao, 2018), and statins only reduced the development of early stent thrombosis in patients with high hsCRP levels (Luan and Yao, 2018); and activated platelets, apoptotic macrophages, and microparticles generate mCRP in atherosclerosis and in stent restenosis; all these suggesting CRP participation in stent thrombosis and failure (Eisenhardt et al., 2009; Habersberger et al., 2012).

Although drug-eluting stents seek to avoid intimal hyperplasia through pharmacological inhibition of vascular smooth muscle cell proliferation, *covered stents* pursue to prevent this process by means of a physical barrier made of synthetic polymers like polytetrafluoroethylene and its expanded form. Patients with diseased saphenous vein grafts requiring percutaneous coronary interventions treated with polytetrafluoroethylene-covered stent-grafts showed a greater failure rate than patients treated with bare-metal stents (Stone et al., 2011), and similar studies using polytetrafluoroethylene grafts did not show superiority of the polytetrafluoroethylene-covered stents compared to the bare-metal stents with respect to restenosis or major adverse clinical events (death, myocardial infarction, and target lesion revascularization) (Ichihashi et al., 2019). All synthetic polymers can develop severe inflammation and thrombosis associated with stent occlusion (Ichihashi et al., 2019). Although surgical revascularization using non-biodegradable polymer devices made of polytetrafluoroethylene, Gore-tex, and polyester (Dacron) has shown to be effective in replacing large-diameter vessels, when used as small-diameter vascular grafts, they were commonly complicated by thrombotic occlusions (Roll et al., 2008). No clear advantage of one over the other has been shown among polytetrafluoroethylene and Dacron prosthetic materials for peripheral vascular surgery (Roll et al., 2008), although it has been proposed that antibiotic impregnated Dacron endovascular stent grafts could be used for aneurysm repair, a technique particularly useful under urgent and unstable clinical situations (Hennessey et al., 2019). All these synthetic materials are limited by their lack of hemocompatibility that can lead to severe inflammation and thrombosis associated with the occlusion of the stents. The need to overcome complications such as thrombosis and reocclusion that impair the long-term performance of covered stent devices leads to the idea of using biological-based materials to enhance device capabilities to support endothelialization (Ichihashi et al., 2019). Tissue-engineered vascular grafts embedded with cells to produce a biomaterial leading to physiological remodeling could lead to the control of serious complications in vascular surgery (Carrabba and Madeddu, 2018). Numerous approaches have been used to produce tissue-engineered vascular grafts, which include scaffold-based methods (using synthetic and natural polymers), decellularized natural matrices, and tissue self-assembly processes, but irrespective of the approach used, how to produce an ideal graft and achieve graft integration still must be improved (Pashneh-Tala et al., 2016). The idea of successfully developing a multilayered vascularized graft with adequate biological and mechanical properties that could be implanted in humans in the future could facilitate vascular remodeling and patency (Singh et al., 2019).

#### Endografts

With the advent of endovascular techniques, endovascular aneurysm repair has largely replaced open surgical repair for anatomically suitable abdominal aortic aneurysms. However, approximately one-third of patients presenting with abdominal aortic aneurysms are deemed unsuitable for conventional endovascular aneurysm repair because of anatomic constraints most often related to proximal neck anatomy (Yoon, 2019). Endografts are devices placed in the inner side of the thoracic aorta that provide support for a weakened artery. In patients undergoing endovascular aneurysm repair, the post-implantation syndrome, characterized by fever and inflammation, occurs frequently. The origin of post-implantation syndrome is not known, but graft composition and the formation of an acute thrombus may play a role (Voûte et al., 2012). The kind of fabric that is utilized in producing endovascular stent grafts may play a role in the development of post-implantation syndrome, detected through an increase in body temperature following the operation and an increase in serum CRP (Voûte et al., 2012). Implantation of expanded polytetrafluoroethylene-based endografts shows less inflammation compared to woven polyester grafts. It has been demonstrated that endovascular aneurysm repair has lower morbidity and mortality than open surgical abdominal aortic aneurysm repair early postoperatively (Kansagra et al., 2018). However, the long-term follow-up showed that both total mortality and aneurysmal-related mortality are superior in endovascular aneurysm repair compared with open repaired patients, probably due to long-term aneurysm sac enlargement in endovascular aneurysm repair. The localization of the aortic aneurysms from the proximal neck to the access vessels may create technical difficulties for the intravascular repair, and devices like fenestrated stent grafts, branched stent grafts, lower profile, and novel sealing designs have the potential of solving several of those problems. As the aortic aneurysm treatment continues to make progress, the patient population that can be treated with an endovascular approach will expand (Kansagra et al., 2018).

#### Valve Replacement

Valvular heart disease affecting over 100,000 people annually in the US has significant morbimortality (Sachdev et al., 2018). The majority of cases employ tissue bio-prostheses to circumvent the risk of using anticoagulation therapies, especially in older individuals. Although conventionally this approach has been contemplated to be a superior option to avoid the use of anticoagulation therapies, more recent analyses have recognized a significant incidence of previously unrecognized thrombosis related to bioprosthetic valve implantation, especially with the more recent appearance of transcatheter aortic valve replacement implantations. The thrombosis of bioprosthetic valves is a major cause of bioprosthetic valve degeneration and frequently has a short-lived presentation leading to delayed recognition and treatment. The risks and benefits of anticoagulation after bioprosthetic valve replacement to avoid thrombosis have been extensively reviewed in the literature, without conclusive results or evidence-based recommendations. Following an episode of bioprosthetic valve thrombosis, lifelong anticoagulation has been recommended. The exponential increased use of transcatheter aortic valve replacement as an alternative to surgical aortic valve replacement in numerous risk groups brought new challenges with regard to valve thrombosis, which have been poorly studied regarding optimal treatment and prevention (Sachdev et al., 2018). Transcatheter bioprosthetic valve replacement has thromboembolism risk in the first few months, and the risk continues thereafter. Valve thrombosis occurs early (<3 months), late (3 months to 1 year), or very late (beyond 1 year) (Dangas et al., 2016). Minimally invasive valve surgery is associated with reduced thrombosis and inflammation (Paparella et al., 2017), reduced interleukin-6, CRP, and fibrinogen levels.

#### Ventricular Assist Devices

Ventricular assist devices are increasingly used in end-stage heart failure patients ineligible for transplantation. Overtime, the ventricular assist device reduced size, cost, and complications, but thrombosis remains a common risk; elevation of lactic dehydrogenase is a useful biomarker of pump thrombosis. Thrombosis incidence in continuous-flow devices (HeartMate II®, Thoratec Corp., Pleasanton, CA) increased substantially from 2.2 to 8.4% from March 2011 to January 2013 (Starling et al., 2014), and pump rotational blood flow seems to play a pivotal role (Radley et al., 2018). Inflammation is a contributing factor to thrombus formation and progression (Radley et al., 2018).

#### Balloon Pumps

The most frequent complication of intra-aortic balloon pumps used in critically ill patients with compromised cardiac function (cardiogenic shock, acute myocardial infarction, left ventricular ejection fraction <35%) is limb ischemia needing thrombectomy with an incidence of 0.9 to 26.7% (De Jong et al., 2018). Thrombosis, a common complication of subclavian intra-aortic balloon pumps (Naqvi et al., 2018), is associated with mCRP production after platelet activation and microparticle generation (McFadyen et al., 2018).

### Devices in the Venous System

#### Venous Catheters

Central venous catheters (tunneled or non-tunneled catheters, implanted ports, peripherally inserted central catheters, and apheresis or dialysis catheters) are broadly used, relatively safe, and cost-effective providing long-term intravenous access for extended antibiotic therapy, chemotherapy, total parenteral nutrition, and other applications. Central venous catheter-related thrombosis becomes symptomatic in about 5% of patients (Kamphuisen and Lee, 2012). Central venous catheter-associated intimal damage activates platelets and the coagulation system, leading to local vasoconstriction, platelet adhesion, fibrin generation, and thrombosis, and thrombosed arteriovenous grafts in dialysis patients contribute to chronic inflammation through increased tumor necrosis factor-α, CRP, and interleukin-6 (Achinger and Ayus, 2019).

#### Vena Cava Filters

Inferior vena cava filters used in venous thromboembolism patients with contraindications to anticoagulation reduce pulmonary embolism but have increased long-term risks of recurrent deep vein thrombosis and inferior vena cava filter thrombosis (Andreoli et al., 2016). Inferior vena cava filter thrombosis rates range between 1.6 and 33% (Andreoli et al., 2016). Permanent placement of inferior vena cava filters associates with significantly higher deep vein thrombosis overtime. Inferior vena cava filter thrombosis may be related to increased baseline risk for thromboembolism, filter emboli entrapment, deep vein thrombosis propagation from lower extremities, or filter's inherent thrombogenicity as a foreign body (Andreoli et al., 2016). Inferior vena cava filter placement was associated with increased 30-day mortality (Turner et al., 2018). By exposing foreign materials, inferior vena cava filters can develop thrombosis and inflammation following the generation of microparticles bearing mCRP (McFadyen et al., 2018), tissue factor, and other proinflammatory molecules. Table 2 summarizes the thrombosis and inflammation challenges and the biomaterial design strategies of arterial and venous medical devices.

**Table 2:** Summary of thrombosis/inflammation challenges and biomaterials design strategies of medical devices.

**Arterial devices**	**Biomaterial design strategies**	**Challenges/complications**	**Future developments**
Coronary artery bypass grafting	Internal thoracic artery/saphenous vein grafts	Uncontrolled chronic inflammation/graft atherosclerosis/thrombosis	Tissue-engineered vascular grafts
Stents	Bare metal/drug eluting/covered	Chronic inflammation/restenosis/early, late and very late thrombosis	Endothelialized stents/tissue engineered multilayered vascular grafts
Endografts	Expanded polytetrafluoroethylene endografts for Endovascular aneurysm repair/	Post-implantation syndrome/long-term mortality	Fenestrated/branched stent grafts/lower profile/novel sealind designs
Valve replacements	Tissue bio-prostheses/transcatheter valve replacement	Valve implantation-associated thrombosis/valve degeneration	Minimally invasive valve replacement to reduce thrombosis and inflammation
Ventricular assist devices	Reduced size/continuous flow/pump rotational pump	Pump thrombosis	Devices with reduced inflammation/thrombosis
Balloon Pumps	Subclavian intra-aortic pumps	Thrombosis/limb ischemia	Devices with reduced inflammation/thrombosis
**Venous devices**			
Venous catheters	Long-term intravenous access	Venous intimal damage/Thrombosis/chronic inflammation	Catheters promoting reduced inflammation/thrombosis
Vena cava filters	Filters to reduce venous thromboembolism	Long-term recurrent deep vein thrombosis/inferior vena cava filter thrombosis/inflammation	Filters promoting reduced inflammation/thrombosis

In the past several years, research has been conducted to identify proinflammatory molecules, like CRP and its distinct isoforms native pentameric CRP and mCRP, as markers/mediators of atherosclerosis and other inflammatory diseases, and also as mediators of thrombus formation and growth, major early and late complications of any medical device placement. Since CRP enhances thrombosis after vascular injury and inflammation upregulates the expression of CRP, CRP appears to be a mechanistic link between inflammation and thrombosis (Fay, 2010). Serum native CRP is a pentameric molecule composed of five non-covalently bound globular subunits arranged as a cyclic annular disk, and it can undergo subunit dissociation into individual monomeric units when associating with a cell membrane (Ji et al., 2007). Whereas, serum native pentameric CRP may not affect thrombus growth, mCRP is prothrombotic enhancing platelet deposition and thrombus growth under arterial flow conditions (Molins et al., 2008, 2011).

## Conclusions

Medical devices use will increase as people live longer. The prevailing challenge is *elimination of device thrombogenesis* since it causes failure, patient morbidity, and mortality. More effective and safer antithrombotic and anti-inflammatory agents and less thrombogenic biomaterial surfaces can overcome the problem. More research and development is needed to understand biological processes involved in coagulation at blood-contacting device surfaces. It is likely that biomaterials used in arterial vs. venous devices would be different since processes participating in both blood-contacting surfaces have some differences, e.g., venous low shear rates can lead to regional accumulation of activated coagulation factors and thrombogenesis on a nearby surface, leading to larger thrombi and increasing the risk for developing thromboembolic disease. Until such developments occur, engineers and clinicians need to work together to develop devices that mitigate thrombosis and inflammation.

## Author Contributions

All authors listed have made a substantial, direct and intellectual contribution to the work, and approved it for publication.

### Conflict of Interest

The authors declare that the research was conducted in the absence of any commercial or financial relationships that could be construed as a potential conflict of interest.
